# “Because you just get stuck in this hamster wheel” - experience and narratives of work-family conflicts among patients in psychosomatic rehabilitation

**DOI:** 10.1186/s40359-026-05615-x

**Published:** 2026-09-21

**Authors:** H. Bindels, H. Miluczenko, M. Bethge, M. Schuler

**Affiliations:** 1 Bochum University of Applied Sciences, Department of Nursing, Midwifery and Therapy Sciences; Gesundheitscampus 6-8, 44801 Bochum, Germany; 2 University of Luebeck, Institute of Social Medicine and Epidemiology; Ratzeburger Allee 160, 23562 Lübeck, Germany

**Keywords:** Work-Family Conflict, Burnout, Affective disorders, Psychosocial burden, Illness narratives, Causal attributions, Qualitative analysis, Patient-centered care

## Abstract

**Background:**

Workplace problems are among the most common reasons for entering psychosomatic rehabilitation (PR). Parents with mental disorders (MD) often face additional caregiving demands alongside combined work and family responsibilities. However, this issue has received little attention in rehabilitation research so far. This so-called Work–Family Conflict (WFC) is associated with depression, anxiety, and other mental health conditions, yet little is known about how patients with MD experience and interpret these conflicts.

**Methods:**

We conducted a secondary qualitative analysis of 32 semi-structured interviews with parents with MD undergoing inpatient PR in Germany. Using a typifying content analysis approach, we combined deductive and inductive category formation to examine recurring ways in which these patients experienced and internally processed WFC. In addition, we developed narrative case summaries to explore how participants link work-related stress, family demands, and their MD to the decision to seek rehabilitation.

**Results:**

We identified eight empirically derived intrapersonal manifestations of WFC, e.g. *Self-Sacrifice, Invisible Loneliness*, and *Threat to Self-Identity*. These manifestations offer an experiential perspective on WFC, capturing recurring patterns of thoughts, emotions, and behaviors. In addition, 13 distinct narrative scenarios emerged, illustrating the diverse explanatory relationships participants constructed between work-related stress, family demands, MD, and their need for rehabilitation. While several patterns overlapped with symptoms of depression or burnout, patients framed them as distinct experiences that captured their everyday struggles with balancing roles.

**Conclusions:**

WFCs represent a clinically relevant dimension of psychosocial burden in parents with MD undergoing PR. Explicitly addressing WFC in clinical assessment and treatment may improve case formulations, validate patients’ lived experiences, and inform individualized rehabilitation strategies. Recognizing WFC as a distinct source of distress may therefore enhance patient-centered psychosomatic care. Future longitudinal research may clarify how WFC relates to rehabilitation outcomes such as symptom reduction, return to work, and quality of life.

## Background

Mental disorders (MD) pose a major public health challenge worldwide [1]. In Germany, they account for a substantial share of sickness absence and are the leading cause of disability pensions [2]. National estimates indicate that about one third of adults meet the criteria for a MD within a year [3].

Inpatient psychosomatic rehabilitation (PR) is a well-established treatment modality in Germany for individuals with mental and psychosomatic disorders. It addresses a broad range of diagnoses, including affective, neurotic, stress-related, and somatoform conditions [4]. Grounded in the biopsychosocial framework of the International Classification of Functioning, Disability and Health, PR places particular emphasis on patients’ real-life contexts, especially in relation to *activity* and *participation* [5]. This approach underscores the importance of understanding mental health conditions within their social environments, particularly in relation to key life domains such as work and family.

While work-related stressors have long been a focus in rehabilitation research and are integrated into therapeutic frameworks - e.g., through work-oriented group therapy or medical-vocational rehabilitation programs [6, 7] - family-related factors have received considerably less attention. Nevertheless, previous research has identified family-related contextual factors as relevant sources of distress among patients in PR[8]. However, the intersection of work-related and family-related strain has rarely been examined in the context of PR. This gap is noteworthy given that a large proportion of PR patients are in the so-called “rush hour of life”, a life phase characterized by the simultaneous demands of career progression and family responsibilities [9, 10]. The co-occurrence of work and caregiving stress may be a critical risk factor for the onset, maintenance or exacerbation of MD [11].

In disciplines such as occupational psychology and sociology, the phenomenon of work-family conflict (WFC), understood as the incompatibility of demands from work and family life, has been extensively studied. Foundational work by Greenhaus and Beutell [12] defined three primary types of conflict at the work-family interface: time-based conflict, which occurs when time devoted to one role limits participation in the other; strain-based conflict, in which strain arising in one role impairs functioning in the other; and behavior-based conflict, which occurs when behaviors expected in one role are incompatible with those required in the other. Later studies distinguished between ‘work interfering with family’ and ‘family interfering with work', highlighting the bidirectional nature of these conflicts [13]. Research over the past decades has consistently demonstrated that the interaction between job and family demands can have significant implications for psychological well-being. Findings show that WFC is associated with increased risk of mood disorders, anxiety, and substance dependence [14], underlining its potential as a serious mental health concern. More recent studies have further emphasized the moderating role of contextual factors such as social support, organizational structures, and role expectations, in shaping the extent and impact of WFC on mental health [15–17]. Furthermore, WFC has also been examined as a mediating factor between job demands und employee mental health [18].

However, previous studies have primarily drawn on samples of psychologically healthy employees, often recruited from corporate or academic settings. It is unclear whether the existing conceptual categories are sufficient to capture the full comprehensiveness of WFC in individuals with MD - particularly those with caregiver responsibilities. Furthermore, most studies relied on quantitative research designs. While such approaches can identify general patterns, they often fall short in capturing the complexity and nuance of individual experiences. This is especially critical in the context of MD, where subjective experience of stress and role conflict may maintain or intensify the situation. In this regard, individual narratives and interpretations offer valuable insights into the lived experience of illness and are increasingly recognized as vital components of patient-centered care and psychosocial treatment approaches [19].

To our knowledge, no studies have specifically examined WFC in clinical samples of patients receiving treatment for MD. Consequently, little is known about how they experience and make sense of such conflicts, how these are situated within personal narratives of illness and recovery, and what role WFC might play in the context of PR. A closer examination of these subjective perspectives could help to clarify the significance of WFC for these patients and explore its relevance as a topic within rehabilitation research more broadly. A qualitative approach is particularly suited to addressing these gaps, as it allows for an in-depth exploration of subjective meaning-making while also focusing on a population that has thus far received little attention in WFC research.

This study addresses the following research questions:

1. How do patients in PR experience conflicts at the intersection of work and family in their everyday lives?

2. How are these conflicts reflected in patients’ subjective narratives about their need for rehabilitation?

## Methods

### Context and qualitative approach

The present work is a secondary analysis based on data gathered as part of the project ‘Family-oriented Rehabilitation for Adults with Mental Disorders” (FER; DRKS00033973; further description provided in the Online Supplement (OS)). The data comprises 32 semi-structured qualitative interviews carried out between May and October 2024 with patients who have children and were undergoing regular PR. Participants were selected because they belonged to the target population of the proposed family-oriented rehabilitation approach; however, they did not receive FER as an intervention. Rather, the interviews formed part of a needs assessment aimed at exploring patients’ hopes, expectations, and concerns regarding such an approach. [20]. Accordingly, the interview guide focused mainly on issues related to family life (English version provided in the OS). The opening question - *“You are currently undergoing rehabilitation at Klinik Höhenried. How did this come about?”* - was designed to allow for maximum openness and did not specify whether participants should refer to personal circumstances or a medical diagnosis. Notably, the topics of work and work-family compatibility emerged repeatedly across all interviews, even though participants were not directly asked about WFC. As a result, this thematic complex was identified and explored as a secondary line of inquiry through a separate analytical process. In doing so, we adopted a narrative-analytical approach to trace the connecting stories of MD and individual life situations. It is important to note that both interviewers (HB & HM) have healthcare backgrounds and were accustomed to confidential patient conversations; however, they had no prior disciplinary grounding in the social science field of work-family intersection, which shaped the openness to unexpected themes. During analysis and interpretation, the findings were continuously examined in relation to the WFC literature, and different interpretations were discussed within the interdisciplinary research team. This team included a psychologist and rehabilitation researchers with relevant expertise in psychosomatic and work-oriented rehabilitation, psychotherapy, and qualitative health research. In addition, HB engaged with current research and scholarly discourse through the Work and Family Researchers Network (WFRN).

### Sampling

Comprehensive information on the sampling strategy, inclusion and exclusion criteria, and data collection processes of the primary study is provided in the OS. The sample consisted of 32 rehabilitation patients (20 women and 12 men), aged between 26 and 57 years. Self-reported reasons for PR included depression, burnout, exhaustion, and anxiety disorders. All participants had between one and four children living in the same household, with child ages ranging from infancy to 25 years. 30 out of 32 interviewees were employed at least part-time, with 13 on sick leave and one person on parental leave. Additional demographic characteristics are presented in Table 1.

**Table 1:** Participant characteristics

	Number / Mean (SD)
**Age (years)**	43,1 (7,9)
**Sex – female/male**	20 / 12
Number of children	
1 child	13
2 children	14
3 children	3
4 children	2
**Family status**	
Married / in a relationship	22
Divorced / seperated (shared custody)	7
Single parent	3
**Employment status**	
Full-time	19
Part-time	11
On sick leave	13
On parental leave	1
Unemployed	2
**Working conditions**	
Shift work	6
Office Job	14
Physically demanding work	9
Social or care-related work	9
**Diagnoses**	
F.32/F.33 Depression	19
F.43 Adjustment Disorder / PTSD	6
F.45 Somatoform Disorder	4
F.41 Anxiety Disorder	3

### Data processing and analysis

All interviews were recorded with Adobe Audition (Adobe Inc., San Jose, CA, USA) or Zoom (Zoom Video Communications, Inc., San Jose, CA, USA), transcribed with noscribe [21], and subsequently reviewed, edited, and anonymized by trained research assistants. To answer the first research question, the interview material was analyzed using qualitative content analysis following Schreier’s toolbox model [22]. We initially used a mixed concept-driven and data-driven strategy of category development. Time-, strain-, and behavior-based conflict were derived from the established WFC model [12] as concept-driven main categories. We explicitly chose not to differentiate between ‘Work Interfering with Family’ and ‘Family Interfering with Work', since maintaining this distinction would not have reflected the way participants constructed their narratives. Within each of the three dimensions, subcategories were developed inductively to differentiate how the respective forms of conflict were experienced in the study population. Relevant passages were assigned to existing subcategories or, where their content was not yet represented, used to establish a new subcategory. During the analysis, however, this theoretically structured coding frame proved insufficient to capture the recurring configurations found in participants’ accounts. We therefore reconsidered the initial coding frame and analyzed the relevant material again using a data-driven category-development strategy, without retaining time-, strain-, and behavior-based conflict as the organizing main categories. By comparing and grouping recurring patterns across the material, eight categories of intrapersonal manifestations were developed. These eight categories constitute an alternative organization of the material rather than an additional hierarchical level beneath the original coding frame. Time-, strain-, and behavior-related aspects remain embedded within them and may occur together within a single intrapersonal manifestation. The final category system therefore reorganizes the theoretically informed initial coding in a way that more closely reflects the configurations found in participants’ accounts.

To address the second research question, a separate analysis was conducted using typifying content analysis [23], independently of the category development for the first research question. The open initial interview question prompted participants to describe how they understood their pathway to PR. For each interview, a structured case summary was prepared that condensed the participant’s subjective assumptions about the relationships between work-related stress, family-related stress, and their MD. The summaries retained the explanatory sequence constructed by the participant rather than imposing a predefined causal order. These case summaries were subsequently translated into schematic configurations. This procedure resulted in 13 distinct scenarios representing different configurations of the subjectively attributed relationships between work-related stress, family-related stress, and MD. Further details on the translation of the case summaries into schematic configurations, the determination of scenario boundaries, and illustrative examples are provided in the OS.

Accordingly, the eight intrapersonal manifestations and the 13 scenarios, including their broader typological grouping, are presented as the principal findings addressing Research Questions 1 and 2, respectively, whereas the underlying coding frame and structured case summaries served as analytic tools.

Data analysis was conducted in MAXQDA (VERBI Software. Consult. Sozialforschung GmbH, Berlin, Germany), with all materials in German; selected excerpts were translated into English for this article. We followed the SRQR guidelines for reporting qualitative research [24].

## Results

1 – Conflicts at the intersection of work and family

The interview material revealed a variety of conflicts at the intersection of work and family. Some accounts could be situated within the established dimensions of WFC. For example, participants reported family-related burdens, such as caring for ill relatives, that negatively affected their work performance, reflecting strain-based conflict. Conversely, work-related demands such as overtime or shift work interfered with family life, reflecting time-based conflict.

“And when my full-time job is over, I go straight to the second job, and I get home around nine or ten or so. Quick shower, barely any time with my son and my wife.” P05

A central finding of our analysis is that the established categories of WFC are insufficient to fully capture the conflict experience in this specific population. Participants primarily described intrapersonal tensions, i.e. internal struggles carried out within the self. These were not experienced as episodic, but rather as structural and persistent and may indicate a more chronic dimension of WFC. Based on the interview data, these tensions were clustered into eight ‘intrapersonal manifestations of WFC’: *External Control, Excessive Performance Demands, Self-Sacrifice, Uncontrolled Boundary Blurring, Moral Conflicts, Invisible Loneliness, Existential Anxiety* and *Threat to of Self-Identity* (see detailed descriptions and quotes in Table 2). The manifestations are not mutually exclusive. In many interviews they co-occurred, interacted, and/or reinforced each other. Each manifestation is accompanied by characteristic emotional states and behavioral reactions (e.g., affective responses, action motives, such as ‘protecting the family’).

“And I was also very (..) emotionally very affected. I would either cry quickly (..) or get really angry. […] Sometimes my co-workers got the worst of it, but especially my family. (..) The very people you’re trying to protect and not hurt.” P07

**Table 2:** Intrapersonal manifestations of WFC

Name	Description	Quote
External Control	Combining work and family feels hardly manageable in everyday life Particularly pronounced in “dual earner couples” and single parents: daily life mainly consists of organizing and coordinating Living on “autopilot” or in function mode Feeling of loss of autonomy Loss of self-determination Feeling dependent on needing help	*“It all felt like—it had nothing to do with living anymore. It was really just functioning, ticking things off.” P19*
Excessive Performance Demands	Heavy workload in both domains (work and family) Feeling pressure from responsibility Having to fulfill multiple roles and expectations Demand to be good at everything "The ideal becomes a compulsion" No direct questioning of identity, but constant performance pressure	*”… suddenly you carry the burden of having to be the best friend. The best sexual partner. The most trusted one, the provider, the dad. The most reliable colleague. Suddenly there are so many roles—I think you simply can’t fulfill them all. Because it’s just too much, which is also very depressing for oneself, yes. (…)” P26*
Self-Sacrifice	Sacrificing oneself for family or work Constant availability for others Focus solely on others’ needs No time for oneself: hobbies, breaks, or time alone are entirely missing Neglecting or not even recognizing one’s own needs Classic example: mother as “family manager”, taking care of all organizational and emotional tasks	*“I normally work all day. I actually have no time for myself. I take care of, save the whole environment, including my mother-in-law and everything else. (…) And actually have no time for myself.” P10*
Uncontrolled Boundary Blurring	Feeling of loss of control: professional and family issues mentally overlap and can no longer be separated voluntarily Lack of recovery—cognitively, emotionally, and physically Constant (unsuccessful) attempts to compensate Mental load: constant circling of thoughts, anticipation, and organization of tasks Overload due to simultaneous demands (“vicious circle”)	*“Because I had too much in my head. My job, then with my son—graduation, will he make it, looking for an apprenticeship. And somehow it was just too much.” P04*
Moral Conflicts	Negative emotional reaction from the feeling of violating (others) basic needs Toward family: guilt due to lack of time or being overwhelmed In relation to work less present, more tied to performance pressure Shame over one’s own reactions and feelings, e.g., calling one’s children a “burden” Deeper shame linked to identity and self-worth	*“Because of course, you do feel guilty, right. Or you’re a bit ashamed. (.) That it turned out this way, that maybe you’re not the strong man you were supposed to be. (..) That’s definitely part of it too.” P26*
Invisible Loneliness	Lack of emotional support: burdens (e.g., job-related) are not sufficiently buffered Partner/family shows little understanding for work-related stress Not being “seen” or perceived as a person Experience of ongoing emotional distance Loneliness Tends to affect the family context (high expectations)	*“But it’s just (.) no real satisfaction—that you might be able to talk about something that came to mind, or something that happened at work, or anything (…). There is someone there, but you feel alone.” P06*
Existential Anxiety	Dilemma between burden and necessity of work Feeling of having no alternatives: work as an essential condition for family stability Financial responsibility as a constant stress factor Worry about maintaining the current standard of living Fear of not being able to provide enough for the family Not being able to “afford” being sick Fear of loss on multiple levels, e.g., fear of social decline, especially concerning the family	*”(…) that I no longer want to work full time, to have more time for my daughter again and not be so stressed here. But I’m afraid it’ll be financially difficult, because I’m a single parent, and the mother isn’t required to pay child support—she couldn’t anyway.” P28*
Threat to Self-Identity	Feeling torn between role images Typical thoughts: “I always wanted to be a good mother”; “My work used to motivate me. Why does it feel different now?” Questioning one’s own abilities and identity No longer living up to one’s own standards Discrepancy between ideal image and current experience Conflict between (former) self-image and current reality	*“My whole life I was always very strong and many admire me for that. And so (…) it took me a long time to realize that I actually need help, that I have to change something.” P17*

**Fig. 1:**
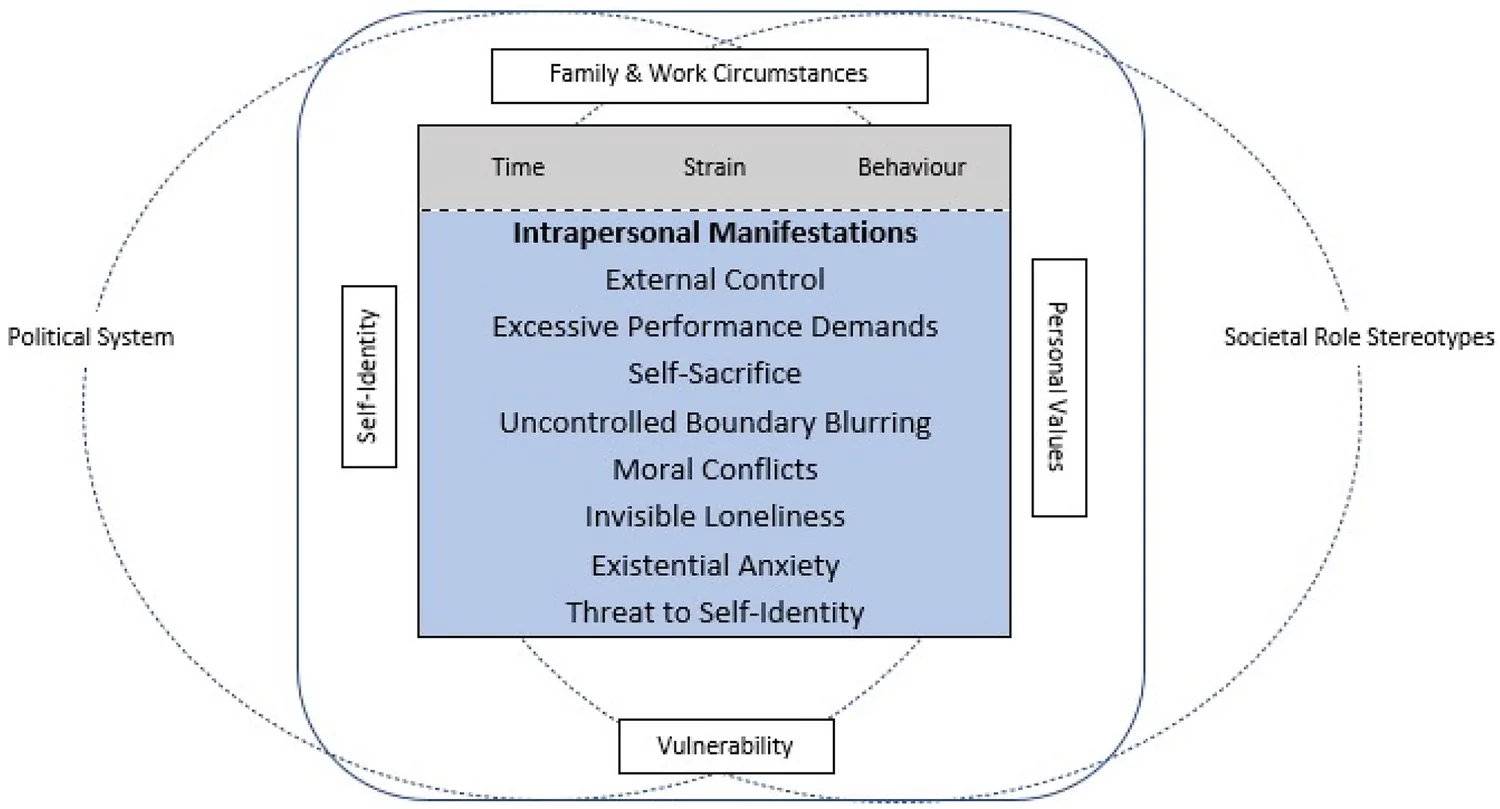
Extended model of WFC in individuals with mental health conditions

The empirically derived intrapersonal manifestations describe recurring ways in which patients experience and process WFC in their thoughts, emotions, and behavior. Rather than extending the established time-, strain-, and behavior-based dimensions of WFC, they provide an experiential perspective on the same phenomenon. Figure 1 situates these manifestations within the established dimensions, which form the conceptual background and may be reflected across several manifestations simultaneously. The model is further embedded in personal, family-related, and work-related circumstances and framed by broader sociocultural conditions, including systemic contexts and gender-specific role expectations. These contextual elements were not derived through the inductive analysis but were included to situate the empirical findings within the broader conditions that may shape how WFC is experienced and managed.

2 –Relation to subjective needs for rehabilitation

**Fig. 2:**
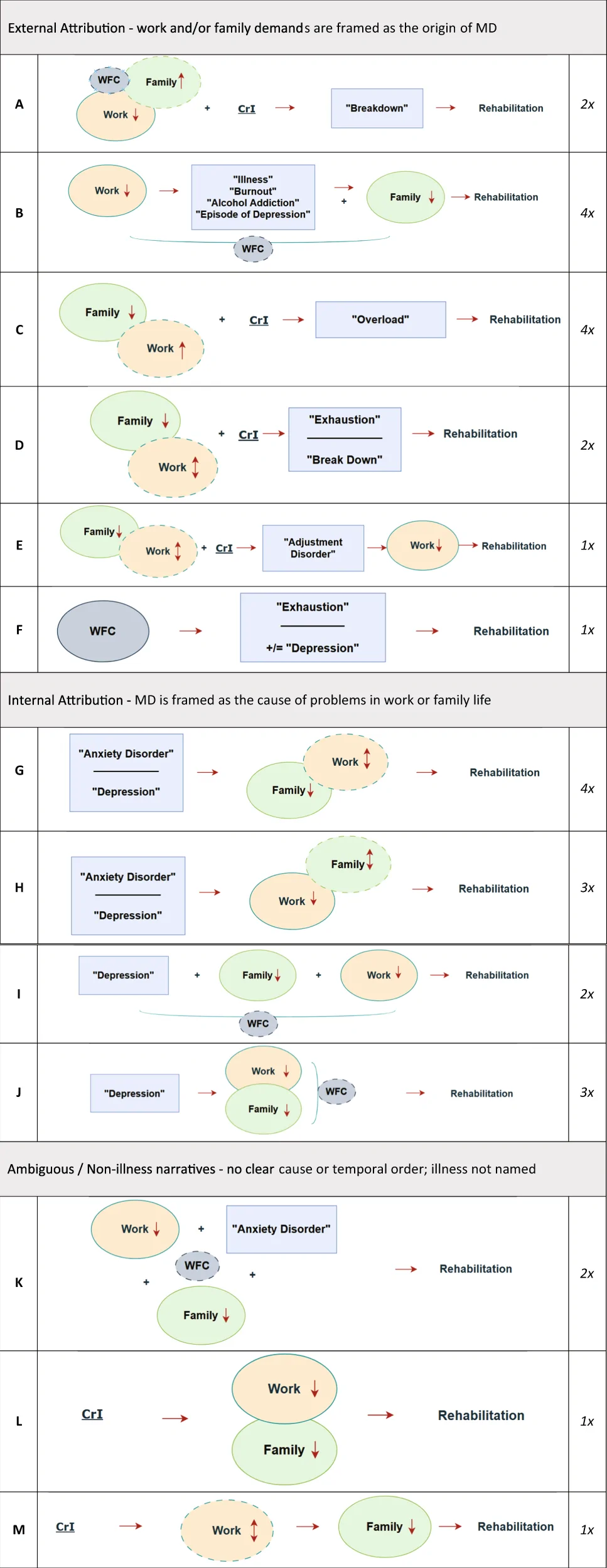
Subjective narratives of work, family and mental disorder

From the 32 individual case summaries, we identified 13 recurring scenarios (Fig. 2) illustrating how participants made sense of their illness biographies through narratives about their perceived need for rehabilitation. All scenarios share the same core elements, which emerged spontaneously in the narratives: family, work, MD (or some form of psychological distress), and in some cases a sudden critical life event. These elements differ in their sequence and in how they are linked to one another, such as through causal (→) or additive (+) relationships. Work and family were described as either burdensome (↓), supportive (↑), or both (↕). Participants perceived the interaction between the elements as dynamic and processual. Although WFC was not always explicitly named, every participant reported experiencing it. This implicit tension is reflected in the close positioning of the work and family ellipses in Fig. 2. In those scenarios where WFC is shown as a discrete element, interviewees articulated the connection in their own words. The rectangular blue boxes reproduce verbatim the words participants themselves used to label their mental health condition. Across all cases, the three domains - work, family, and MD - were described as interconnected and mutually influential, yet differing in their relative weighting and configuration. Visually, dominant domains are shown as solid-line ellipses, less prominent ones as dashed.

Further, the scenarios differ in how participants attributed the origin of their problems. Scenarios A-F reflect interpretive patterns where MD is perceived as a consequence of enduring strain or overload in the work and/or family domain. For example, in Scenario C, family-related stress was perceived as the initial trigger (↓), while work initially functioned as a stabilising resource and a source of self-efficacy (↑). This equilibrium held for some time, until a sudden critical incident (CrI) disrupted it. The resulting sense ‘overload’ led to the pursuit of rehabilitative care.

“The only thing that ever really worked was going to work. […] In the end, I kept myself constantly and endlessly busy so I wouldn’t have to face it [the family situation]. […] And if it hadn’t been for that illness [Covid-19], I’d probably still be there. (.) Because you just get stuck in that hamster wheel.” P19

Other participants identified their MD as the primary cause of both work and family-related challenges (No. G-I). Scenario J, for example, involved a clear diagnosis of depression that negatively impacted both job performance and family relationships. The interaction of these stressors was experienced and described as a WFC and eventually led to the decision to seek rehabilitation.

“The combination [of work and family] definitely acted as an accelerant [for the depression], giving it more momentum, because the additional burden became very, very heavy over time. (.) Especially on an emotional level.” P027

In most scenarios, participants recounted either an accumulation of stressors across domains or a cascading effect, where initial strain in one area led to difficulties in others. Some narratives align with a summative stress model, in which burdens from work, family, or health build up over time until a subjective tipping point is reached. Other cases reveal more complex trajectories, in which causal attributions become blurred. Some participants described a simultaneous interplay of all three domains without assigning a specific causal direction (No. K):

“Yes. (.) That’s why it’s hard to say now: ‘was it really the depression, or was it just the overload you experience [from work and family], so much so that you label it as depression? (..) even though maybe it was just the overload” P15

A general trend among participants who primarily attributed their MD to external factors is the use of nonspecific terms such as “Burnout” or “Exhaustion,” with an apparent reluctance to use formal psychiatric diagnoses. Conversely, when MD was viewed as the origin of difficulties in work or family life, participants were more likely to refer to it using clinical language, such as “depression” or “anxiety disorder.” Scenarios L and M represent special cases, as the participants deliberately avoided any reference to strain or illness in their wording, instead framing a critical life event as the origin of their work and family strain that ultimately resulted in rehabilitation treatment.

## Discussion

This study has two outstanding characteristics: first, it employs a qualitative approach to gain insight in the individual experience of WFC. Second, it focuses on a specific population - individuals with MD and caregiving responsibilities for children. Based on interview data, we identified eight *intrapersonal manifestations of WFC* and embedded them into an extended model. Complementary case analyzes revealed 13 subjective narrative scenarios, illustrating how the domains of work, family, and MD interrelate in shaping individuals’ perceived need for rehabilitation.

The eight intrapersonal manifestations identified in this study provide an experience-oriented perspective that complements the established distinction between time-, strain-, and behavior-based conflict, which may not adequately capture how WFC is subjectively experienced and processed in this clinical population. Only few qualitative studies have explored individual experiences of WFC in depth. Alsarve et al. [25], for example, describes how social norms and expectations produce guilt and a constant mental load, closely related to what we term *Moral Conflicts* and *Uncontrolled Boundary Blurring*. Speights et al. [26] examined emotional responses to WFC, showing that the type and intensity of emotion - often frustration, guilt, or hostility - varied depending on whether the conflict originated in the work or family domain. However, both studies focus on psychologically healthy, middle-class populations and refer to more clearly defined conflict episodes. In contrast, participants in our study rarely referred to discrete conflict episodes, nor did they locate the source of tension in one domain alone. Instead, they described a persistent, reciprocal tension between work and family roles.

Our findings revealed that PR patients experience WFC as an enduring burden, pointing to its potentially chronic nature. Inner conflicts often persisted even after the original external stressors had been resolved. For example, one participant changed his work group to escape mobbing and stress, yet the WFC remained and he still experienced *existential anxiety*. Another defining feature is that the *intrapersonal manifestations* are not exclusive and may be connected to each other. Furthermore, rather than being simply reactions to external stressors, they may themselves become new sources of strain. For instance, avoiding conversations about work-related struggles in order to protect family harmony (*Self-Sacrifice*) can lead to *Invisible Loneliness*. In this way, *intrapersonal manifestations of WFC* may simultaneously function as coping strategies, manifestations, precursors, consequences, or amplifiers of further conflicts. These recursive dynamics are the very embodiment of chronicity and therefore lie beyond the explanatory scope of traditional WFC models, which are typically based on a linear, unidirectional logic (e.g., Work Interfering with Family, Family Interfering with Work [13]; for a more dynamic perspective, see [27]).

Some participants struggled to disentangle the conflict from their MD. This might reflect that several of the *intrapersonal manifestations* - resemble core depressive phenomena. A bottom-up review by Fusar-Poli et al. [28] vividly illustrated how individuals with depression experience their condition. They reported similar feelings, such as guilt, emotional emptiness, loneliness, and a sense of disconnection from the self. What distinguishes the *intrapersonal manifestations*, however, is that they are not as pervasive or deep-rooted. Instead, they reflect the dynamic interplay between strain in two concrete life domains. For example, whereas Fusar-Poli describes feelings of universal guilt without a clear trigger (“feeling guilty for simply existing”), the guilt expressed in our *Moral Conflict* category is contextual, for instance, feeling inadequate as a parent due to work-related tensions. Moreover, the *intrapersonal manifestations of WFC* do not necessarily appear to be pathological in nature. Participants described WFC not as clinical symptoms, but as a legitimate burden or structural strain shaping everyday life. In fact, several participants in our sample also described clear depressive symptoms (e.g., sadness, lack of motivation, insomnia), which they primarily associated with their MD. However, these were mentioned in distinct contexts, suggesting that patients differentiate between WFC and depression. The extent to which depression initiates, sustains, or amplifies the experience of WFC cannot be determined from our data and therefore remains unclear, highlighting the need for further research.

To better understand the *intrapersonal manifestations*, it is helpful to consider how internal psychological mechanisms may shape the experience of WFC. Psychological conflict theories, such as those discussed by Grawe [29], highlight the strain that can arise when individuals are unable to tolerate internal contradictions. When ambiguity tolerance is low, and personal needs, expectations, and external realities begin to diverge, this mismatch might contribute to the development of mental health conditions. This points to the contextual factors outlined in our model (Fig. 1), including personal values, individual vulnerability, and self-identity, which may influence how individuals process and regulate ambiguous emotional experiences.

WFC also shows some conceptual overlap with the clinical notion of burnout. Some interviewees even described themselves as “burned out” to capture their condition. Etiological models (e.g., [30, 31]) describe burnout as resulting from an interplay between structural stressors (e.g., working environment) and individual vulnerability, particularly the mismatch between demands and personal coping capacities. This resonates with our findings: although external demands are relevant, both WFC and burnout tend to evolve into persistent, internalized strains that outlast their original triggers. Moreover, the twelve burnout stages [32] show content-related similarities to the *intrapersonal manifestations* (e.g., Phase 3: ‘neglecting needs’ and *Self-Sacrifice*; Phase 5: ‘revision of values’ and *Threat to Self-Identity*). In this sense, WFC may be seen as extending the concept of burnout by adding the dimension of family-related strain and emphasizing its interdependency.

However, although WFC shares certain features and overlaps with depressive and burnout-related symptoms, it may be more useful to regard it as a distinct phenomenon within the context of PR. Recognizing *intrapersonal manifestations of WFC* may complement depressive or stress-related diagnoses. Furthermore, to better capture the diversity of WFC experiences, future research may conceptualize WFC along a continuum - ranging from isolated, episodic conflicts to more persistent, chronic forms. Based on the present findings, we hypothesize that the WFC experiences represented in our clinical sample may be situated toward the chronic end of such a continuum: Participants reported long-lasting, seemingly intractable conflicts despite repeated attempts to resolve them through various strategies. In contrast, much of the existing WFC research appears to focus more strongly on the other end of the continuum, emphasizing acute, situation-triggered conflicts [33, 34]. This conceptualization raises several relevant research questions: For example, do existing diagnostic tools (c.f., [35, 36]) adequately capture the individual dynamics of WFC in a clinically useful manner or should they be expanded to better reflect typical intrapersonal manifestations and coping patterns? Are such manifestations of WFC also to be expected in “healthy” individuals if the strain persists long enough or do these manifestations occur in clinical populations only? How can the progression toward chronicity be identified and potentially prevented?

The personal narratives may provide valuable insights related to these questions and may have particular clinical relevance. It is widely acknowledged that narratives are shaped by subjective experiences, emotions, and the need to find meaning. People use storytelling to process their feelings, reconstruct their identities, and integrate illness into their life stories. The narrative process itself can be therapeutic, supporting personal growth and resilience [37]. In our study, conflicts at the work-family interface emerged as participants’ primary explanatory framework, shaping both their understanding of their condition and their perceived need for rehabilitation.

A notable aspect of our results is the fact that, despite the limited number of elements comprising the narratives, a total of 13 distinct scenarios could still be identified in a sample of 32 participants. This narrative variety contrasts with the concern raised by Thomsen et al. [38], who highlight the risk that individuals may internalize a constraining ‘master narrative’ of MD - a dominant identity script that may turn negative and exert a totalizing influence on how individuals make sense of their MD (i.e., as a result of trauma, adversity, and vulnerability, often followed by relapse and impaired functioning in socially valued areas). In our sample, however, we found no evidence for one dominant explanatory pattern. Instead, there was considerable variability in the distribution of scenarios, indicating a high degree of narrative differentiation. These illness narratives can actively be integrated into therapeutic work, as they offer valuable insight into patients’ implicit beliefs, emotional tensions, and coping strategies. For example, in their ‘Metamorphic Model’ of narrative identity and illness recovery, Thomsen et al. [38] propose that therapists can take on the role of co-authors, helping individuals reshape constraining narrative identities.

Our findings resonate with this approach, suggesting that fostering narrative flexibility may be particularly relevant in the context of PR. Although the patterns identified here are neither exhaustive nor definitive, a preliminary trend suggests two narrative positions: 1. External attributions - scenarios that locate the origin of distress primarily in work or family systems - tended to co-occur with feelings of external control, excessive performance demands, self-sacrifice, and uncontrolled boundary blurring (i.e., the first four *intrapersonal manifestations*). Individuals in this group often described their condition using non-medicalized terms such as “burned-out,” “exhausted,” or “overwhelmed”. 2. Internal attributions - scenarios regarding which the MD itself is the precipitating factor - more often coincided with moral conflicts, invisible loneliness, existential anxiety, and threat to self-identity (the last four manifestations). These individuals were more likely to use medicalised language and refer to psychiatric diagnoses when describing their situation. However, future studies with larger sample sizes should examine whether specific occupational and family contexts systematically relate to different narrative positions and intrapersonal manifestations of WFC. Further research should also assess whether the observed diversity of narratives can be replicated or whether in larger samples, certain narratives emerge as dominant. Longitudinal designs may additionally shed light on the stability of these narratives and their relations to outcomes such as symptom reduction, return-to-work, and quality of life. Future studies could complement established WFC scales with ecological momentary assessment to capture conflict experiences that are context-dependent and potentially specific to individuals with MD.

Building on the present findings, a deeper understanding of intraindividual WFC experiences may inform intervention strategies. While structural changes (e.g., workplace childcare) are often recommended to mitigate WFC, evidence of their effectiveness is mixed [39–41]. Recent meta-analytic findings suggest greater benefits from interventions targeting individual resources such as coping strategies [42]. In this regard, PR as a resource-oriented and individualized approach may help interrupt the self-reinforcing dynamics of WFC by strengthening emotional and cognitive resilience (e.g., via cognitive-behavioral techniques). In targeted group or individual therapy, patients could learn communication, boundary-management, and coping strategies tailored to their specific WFC-patterns. Particular attention could be paid to helping patients communicate work- and family-related concerns, needs, and fears with family members and in the workplace. This focus on individual resources should not be understood as assigning responsibility for structurally produced burdens to patients. Workplace conditions, childcare arrangements, financial constraints, gendered divisions of labour, and social-policy factors should likewise be considered in treatment. To date, very few studies have addressed WFC in the context of rehabilitation. One exception is the work of Bethge and Borngräber [43], who examined WFC among women with chronic musculoskeletal disorders. They argue that modern rehabilitation should broaden its focus beyond traditional occupational stressors to include family-related demands as relevant contextual factors. Although their study focused on a different patient group, their call to integrate family-related stressors into rehabilitative care aligns with the implications of our findings.

### Limitations

This study has several limitations that should be considered when interpreting the results:

First, this is a secondary analysis of qualitative interview data collected as part of the project FER which evaluates the implementation of a family-oriented rehabilitation concept. The original interview guide was not designed to specifically explore WFC, but rather focused on patients’ hopes, expectations, and fears regarding family involvement. Therefore, integrating questions to explore WFC more directly might have led to other results. However, this can also be seen as a strength of the study: Patients did not reflect on questions about WFC, but WFC emerged spontaneously, without direct prompting, which might have reduced influences of social desirability. Nevertheless, the context-specific sample (already prepared to talk about family issues) may have contributed to this emphasis.

Second, the qualitative data were collected from patients undergoing rehabilitation at one single clinic in Germany. As all participants were parents recruited within a family-oriented project, individuals with pronounced WFC may have been more likely to participate. It is uncertain whether individuals with MD who do not have children experience WFC in a similarly pronounced or multifaceted way. Moreover, it remains unclear whether the diagnostic distribution observed in this sample of parents is representative of the broader population of patients undergoing PR. Together, these sample characteristics may limit the representativeness and generalisability of our findings.

Third, the analysis followed an exploratory approach and does not claim to be exhaustive. In particular, the scenarios related to the second research question reflect highly individual narratives. These were refined through an iterative process and ultimately reduced to 13 core scenarios consisting of the same elements. A larger sample would likely yield additional scenarios with further elements and subjective interpretations of causality.

## Conclusion

For parents with MD, WFC plays a central role in the emergence of subjective rehabilitation needs. Their narratives to explain these needs reveal a complex interplay between work demands, family roles, and the illness process. This study identified eight typical *intrapersonal manifestations* that present as enduring, internal conflicts and may themselves influence the dynamic of WFCs. While several of these manifestations resemble symptoms of depression and burnout, we propose that they should be viewed as conceptually distinct, informing about significant sources of psychological strain. Clinically, explicitly naming and exploring WFC may enhance case formulations, support individualised interventions, and validate patients’ lived realities. For research, a clearer conceptualization of WFC offers promising insights into how the pressures of work and family life shape symptom trajectories and recovery processes.

### Online Supplement (OS)

#### Project description: FER

The aim of the project *Family-Oriented Rehabilitation for Adults with Mental Disorders* (*Familienorientierte Erwachsenenrehabilitation bei psychisch Erkrankten*, FER) is to develop and implement a family-oriented rehabilitation program that systematically involves partners and children in the therapeutic process through modular interventions. The entire process is accompanied by scientific evaluation. The project is funded by the German Pension Insurance South Bavaria (Germany) through the grant program “Innovative Wege zur Teilhabe am Arbeitsleben - rehapro” (Innovative Ways to Participate in Working Life - rehapro), which was initiated by the Federal Ministry of Labour and Social Affairs to find new ways to maintain or restore work participation of people with health impairments by testing innovative services and/or organizational measures.

The first qualitative sub-study of FER explored expectations, hopes, and concerns of potential FER participants [44]. These findings were intended to support program acceptance and facilitate adaptation to the actual needs of the target group. The present analyzes build on this initial study, drawing on its data set for secondary in-depth analysis.

Further details on the study design, study arms, sample size, and evaluation framework are available in the German Clinical Trials Register (https://www.drks.de/search/de/trial/DRKS00033973/details; German and English language). Further information about the project and the participating clinic can be found on the project website: https://www.deutsche-rentenversicherung.de/BayernSued/DE/Ueber-Uns/FER/FER_node.html and the website of the rehabilitation center: https://hoehenried.de/home/abteilungen/psychosomatik/familienorientierte-erwachsenen-reha-fer/ (both German language only).

## Methods

### Conceptual framework

This qualitative study followed the *Quality Criteria of Qualitative Studies in Rehabilitation Research* [45] and complied with ethical standards. Approval was granted by the Ethics Committee of Bochum University of Applied Sciences (reference number: 240105_Schuler).

Semi-structured interviews were conducted with rehabilitation patients and their family members. Each participant took part in a single interview, scheduled at varying time points (before, during, or after the rehabilitation stay) to capture different experiences and levels of knowledge. The interview guide was developed based on relevant literature and the research team’s practical expertise. Narrative prompts were used to encourage openness and elicit rich accounts of family dynamics and everyday life with mental disorders. To minimise bias, the FER concept was not presented in detail prior to the interviews.

### Data collection and sample

Between May and September 2024, participants were recruited from the standard psychosomatic rehabilitation program at Klinik Höhenried. Eligible patients were those with an indication for psychosomatic rehabilitation due to a MD classified within ICD-10 categories F3.x (affective disorders) or F4.x (neurotic, stress-related, and somatoform disorders) and with at least one child (up to 20 years of age) living in their household, reflecting the intended target group for a family-oriented rehabilitation approach. Exclusion criteria were insufficient German language proficiency and psychiatric conditions incompatible with psychosomatic rehabilitation, such as acute suicidality. Adult relatives (18 years and older) were also included to broaden the sample. Recruitment aimed to capture heterogeneity in family constellations (e.g., single-parent households, blended families) as well as diverse socioeconomic contexts.

Recruitment proceeded until theoretical saturation was reached. Potential participants were identified by psychotherapists at Klinik Höhenried. Interested individuals received detailed study information from a research assistant, and written informed consent was obtained. Interviews were conducted either on-site at the clinic or online via video conference, with an average duration of 60 minutes (range: 38–88 minutes).

For the present secondary analysis, only patient interviews were considered; interviews with relatives were not included.

Supplementary details on the analysis for research question 2: Development of case configurations and scenarios

The structured case summaries were translated into schematic configurations representing participants’ subjective explanations of the relationships between work-related stress, family-related stress, and MD. Causal arrows were used when participants explicitly attributed one condition to another or when a directional relationship was consistently established through the temporal and explanatory sequence of their account. By contrast, a plus sign indicated that two conditions were described as jointly contributing to the situation without a clear directional relationship. A domain was designated as predominant when it occupied the central position in the participant’s explanation of the development or maintenance of the MD; domains that contributed to the overall configuration but were not presented as central were marked as secondary. This assessment was based on the meaning and explanatory weight attributed to a domain within the interview as a whole, rather than on the frequency with which it was mentioned. Problems, resources, explicit experiences of work-family incompatibility, and critical incidents or life events were likewise included when participants assigned them a relevant role in their explanation.

Scenario boundaries were determined through systematic comparison of the resulting case configurations. Cases were assigned to the same scenario when they shared the same basic relational structure, particularly regarding the attributed causal direction or additive relationship between work, family, and MD and the predominance of the respective domains. A separate scenario was established when a case showed a substantively different configuration of these relationships. The resulting 13 scenarios therefore represent distinct relational configurations identified across the material rather than individual participants. When several participants shared the same basic configuration, they were assigned to the same scenario despite differences in their specific biographical circumstances or individual stressors. The scenarios were subsequently compared for broader similarities and contrasts and grouped into the final types. For example, one participant stated: “Yes, the trigger was, um, the workplace. The working conditions, they led to the MD.” Considered together with subsequent passages describing effects on family life, this account was condensed into the basic sequence B: “work-related stress → MD → consequences for family life.” The causal arrow thus reflected the participant’s own explicit attribution, whereas the scenario represented an analytical condensation of the explanatory configuration developed across the interview as a whole.

Although the analysis drew on the explanatory sequence constructed by participants, it did not constitute narrative analysis in the stricter methodological sense. Narrative form, linguistic structure, positioning, and the interactional production of the accounts were not systematically analyzed. Instead, participants’ narrated temporal and causal attributions were reconstructed and condensed for the purposes of case comparison, scenario development, and typification.

## Supplementary information

Supplementary Information

Supplementary Information

## Data Availability

The qualitative data generated and analyzed during the current study are not publicly available due to the sensitive and potentially identifiable nature of the interview material. Although participants provided consent for the publication of anonymized excerpts (quotes) in this manuscript, they did not consent to the sharing of complete datasets with third parties. Given the small sample size and the specific clinical context, sharing the full data would not comply with ethical standards or data protection regulations.

## Abbreviations

CrI: Critical incident / critical life event
FER: Family-oriented Rehabilitation for Adults with Mental Disorders
MD: Mental Disorder
PR: Psychosomatic Rehabilitation
WFC: Work-Family Conflict
