# Supplementary material for: “Because you just get stuck in this hamster wheel” - experience and narratives of work-family conflicts among patients in psychosomatic rehabilitation
Source: BMC Psychol. 2026 Sep 21;14:1362. doi: 10.1186/s40359-026-05615-x (PMC13595641; doi:10.1186/s40359-026-05615-x)
Supplement: Supplementary file 1 [file 40359_2026_5615_MOESM2_ESM.pdf]

Topic / function	Question / prompt	Follow-up	Optional
**Access / referral**	You are currently undergoing rehabilitation at Klinik Höhenried. How did this come about?<br><br>Could you describe the key steps that led you to this rehabilitation program?	Who or what first suggested or encouraged the idea of attending rehabilitation?<br><br>What doubts or concerns did you have about taking part in a rehabilitation program?	How long after your diagnosis did this happen?<br><br>What obstacles or difficulties made it challenging to get here?<br><br>Who or what supported you along the way?
**Expectations**	What expectations did you have before beginning the treatment?<br><br>What did you already know about the rehabilitation program before you started?	Did you have specific ideas of what to expect?<br><br>What previous experiences did you have with treatments (e.g., prior rehab, outpatient psychotherapy, etc.)?	How did you learn about the rehabilitation beforehand (for instance: where, when, through whom)?
**Condition/ diagnosis**	What do you know about your condition or diagnosis?<br><br>How do you personally deal with your condition?	When and by whom was the diagnosis made?<br><br>Where or how do you usually get information about your condition?<br><br>Have you developed any personal strategies to cope with it?	How well informed do you feel about your condition? What kind of information do you feel is missing?<br><br>How well do these strategies work for you, and where did you learn about them?<br>Where do you see limits or challenges of these strategies?
**Family roles & dynamics**	Who do you consider part of your family?	How would you describe your role within your family?<br><br>What works well in your family? What could work better?	Which caregivers or key support people are involved in your children’s lives (beyond your immediate family)?

	What does family mean to you?<br><br>What does a “typical day” look like for you and your family?	Who takes on which roles or responsibilities?<br>- Parenting<br>- Household<br>- Organizational tasks<br>- Preschool / School<br>- Employment<br>- Relationship quality / dynamics	
**Openness and communication regarding mental condition**	How openly do you talk about your condition within your family? Who knows about it?<br><br>How does your family deal with your condition? (Partner, children, others)	At what point did your family become involved, and in what way?<br><br>How do you talk about your condition: With your partner? With your children?<br><br>How do you talk about your inpatient rehabilitation stay?<br><br>Who else in your close social circle is involved or aware of your situation?	How are your condition, situation, and emotions discussed within the family?<br><br>What makes it easier or harder for you to talk about these topics with your partner or children?
**Impact on everyday life**	How does your condition affect your daily family life?<br><br>How does your condition affect your work or occupational life?	In which specific areas do conflicts or tensions arise? (childcare, school, leisure, work, finances, partnership, etc.)<br><br>How would you describe your relationship with your employer and colleagues?<br><br>How would you describe the interaction between your work and your illness? (In both directions: How work affects your condition and how your condition affects your work)	How do you handle conflicts within your family?<br><br>To what extent could these issues be addressed as part of a family-oriented rehabilitation program?<br><br>To what extent are you currently (during inpatient stay) involved in work activities, or do you currently have contact with your workplace?

**Support wishes / needs**	How does your family support you in dealing with your condition in everyday life?	How do you experience this support? What do you find helpful, and what do you find burdensome or difficult?<br><br>Are there needs or wishes you do not express to your family? Things you prefer not to discuss with them?<br><br>What additional kind of support would you wish for from your family?	Why? What do you think would happen if you did?<br><br>To what extent could these topics be part of a family-oriented rehabilitation program?
**Family involvement in rehabilitation**	We are currently developing a family-oriented rehabilitation concept. In your opinion, what could the involvement of family members in treatment at the clinic look like?<br><br>In which areas could you imagine a meaningful inclusion of family members?<br><br>When you think about the time after your rehabilitation, how could the strategies and insights you have learned here be applied in your everyday family life in the long term	What advantages do you see in this, both for yourself and for your family members?<br><br>What concerns do you have, both for yourself and for your family members?<br><br>Specifically, what could this look like for your partner? For your children?<br><br>Should all family members be involved? If not, who should (or should not) be included, and why?<br>What do you feel you need right now to make that possible?<br><br>What would you advise future rehabilitation patients as they prepare for or go through their own stay?	What practical or organizational challenges do you foresee if family members are included in the rehabilitation process?<br><br>For example regarding:<br>- Time requirements<br>- Accommodation<br>- Additional costs<br>- Compatibility with work or daily responsibilities<br><br>Under what circumstances would you *not* take part in such a program for yourself? For your family or daily life?
