# Supplementary material for: “Because you just get stuck in this hamster wheel” - experience and narratives of work-family conflicts among patients in psychosomatic rehabilitation
Source: BMC Psychol. 2026 Sep 21;14:1362. doi: 10.1186/s40359-026-05615-x (PMC13595641; doi:10.1186/s40359-026-05615-x)
Supplement: Supplementary file 2 [file 40359_2026_5615_MOESM1_ESM.docx]

**Online Supplement (OS)**

### Project Description: FER

The aim of the project Family-Oriented Rehabilitation for Adults with Mental Disorders (Familienorientierte Erwachsenenrehabilitation bei psychisch Erkrankten, FER) is to develop and implement a family-oriented rehabilitation program that systematically involves partners and children in the therapeutic process through modular interventions. The entire process is accompanied by scientific evaluation. The project is funded by the German Pension Insurance South Bavara, Germany through the grant program “Innovative Wege zur Teilhabe am Arbeitsleben - rehapro” (Innovative Ways to Participate in Working Life - rehapro), which was initiated by the Federal Ministry of Labour and Social Affairs to find new ways to maintain or restore work participation of people with health impairments by testing innovative services and/or organizational measures.

The first qualitative sub-study of FER explored expectations, hopes, and concerns of potential FER participants (Miluczenko et al., 2025). These findings were intended to support program acceptance and facilitate adaptation to the actual needs of the target group.
The present analyzes build on this initial study, drawing on its data set for secondary in-depth analysis.

Further details on the study design, study arms, sample size, and evaluation framework are available in the German Clinical Trials Register (<https://www.drks.de/search/de/trial/DRKS00033973/details>; German and English language). Further information about the project and the participating clinic can be found on the project website: https://www.deutsche-rentenversicherung.de/BayernSued/DE/Ueber-Uns/FER/FER_node.html and the website of the rehabilitation center: <https://hoehenried.de/home/abteilungen/psychosomatik/familienorientierte-erwachsenen-reha-fer/> (both German language only).

### Methods

#### Conceptual Framework

This qualitative study followed the Quality Criteria of Qualitative Studies in Rehabilitation Research (Klemmt et al., 2024) and complied with ethical standards. Approval was granted by the Ethics Committee of Bochum University of Applied Sciences (reference number: 240105_Schuler).

Semi-structured interviews were conducted with rehabilitation patients and their family members. Each participant took part in a single interview, scheduled at varying time points (before, during, or after the rehabilitation stay) to capture different experiences and levels of knowledge. The interview guide was developed based on relevant literature and the research team’s practical expertise. Narrative prompts were used to encourage openness and elicit rich accounts of family dynamics and everyday life with mental disorders (MD). To minimise bias, the FER concept was not presented in detail prior to the interviews.

#### Data Collection and Sample

Between May and September 2024, participants were recruited from the standard psychosomatic rehabilitation program at Klinik Höhenried. Eligible patients were those with an indication for psychosomatic rehabilitation due to a MD classified within ICD-10 categories F3.x (affective disorders) or F4.x (neurotic, stress-related, and somatoform disorders) and with at least one child (up to 20 years of age) living in their household, reflecting the intended target group for a family-oriented rehabilitation approach. Exclusion criteria were insufficient German language proficiency and psychiatric conditions incompatible with psychosomatic rehabilitation, such as acute suicidality. Adult relatives (18 years and older) were also included to broaden the sample. Recruitment aimed to capture heterogeneity in family constellations (e.g., single-parent households, blended families) as well as diverse socioeconomic contexts.

Recruitment proceeded until theoretical saturation was reached. Potential participants were identified by psychotherapists at Klinik Höhenried. Interested individuals received detailed study information from a research assistant, and written informed consent was obtained. Interviews were conducted either on-site at the clinic or online via video conference, with an average duration of 60 minutes (range: 38–88 minutes).

For the present secondary analysis, only patient interviews were considered; interviews with relatives were not included.

#### Supplementary details on the analysis for research question 2: Development of case configurations and scenarios

The structured case summaries were translated into schematic configurations representing participants’ subjective explanations of the relationships between work-related stress, family-related stress, and MD. Causal arrows were used when participants explicitly attributed one condition to another or when a directional relationship was consistently established through the temporal and explanatory sequence of their account. By contrast, a plus sign indicated that two conditions were described as jointly contributing to the situation without a clear directional relationship. A domain was designated as predominant when it occupied the central position in the participant’s explanation of the development or maintenance of the MD; domains that contributed to the overall configuration but were not presented as central were marked as secondary. This assessment was based on the meaning and explanatory weight attributed to a domain within the interview as a whole, rather than on the frequency with which it was mentioned. Problems, resources, explicit experiences of work-family incompatibility, and critical incidents or life events were likewise included when participants assigned them a relevant role in their explanation.

Scenario boundaries were determined through systematic comparison of the resulting case configurations. Cases were assigned to the same scenario when they shared the same basic relational structure, particularly regarding the attributed causal direction or additive relationship between work, family, and MD and the predominance of the respective domains. A separate scenario was established when a case showed a substantively different configuration of these relationships. The resulting 13 scenarios therefore represent distinct relational configurations identified across the material rather than individual participants. When several participants shared the same basic configuration, they were assigned to the same scenario despite differences in their specific biographical circumstances or individual stressors. The scenarios were subsequently compared for broader similarities and contrasts and grouped into the final types. For example, one participant stated: “Yes, the trigger was, um, the workplace. The working conditions, they led to the MD.” Considered together with subsequent passages describing effects on family life, this account was condensed into the basic sequence B: “work-related stress → MD → consequences for family life.” The causal arrow thus reflected the participant’s own explicit attribution, whereas the scenario represented an analytical condensation of the explanatory configuration developed across the interview as a whole.

Although the analysis drew on the explanatory sequence constructed by participants, it did not constitute narrative analysis in the stricter methodological sense. Narrative form, linguistic structure, positioning, and the interactional production of the accounts were not systematically analyzed. Instead, participants’ narrated temporal and causal attributions were reconstructed and condensed for the purposes of case comparison, scenario development, and typification.

**Literature**

Klemmt, M., Heide, M. S., Schwarz, B., Klemmt, C., & Kalski, L. (2024). Qualitätskriterien qualitativer Studien in der Rehabilitationsforschung. *Die Rehabilitation*, *63*(05), 323–329. https://doi.org/10.1055/a-2300-3606

Miluczenko, H., Bindels, H., Schuler, M., Gerlach, D., & Thiller, A. (2025). „Familienorientierte Erwachsenenrehabilitation bei psychisch Erkrankten“ (FER) – Welche Erwartungen und Befürchtungen bestehen seitens der Rehabilitand*innen? *Deutscher Kongress Für Rehabilitationsforschung: Mensch Trifft Maschine - Digitale Chancen in Prävention Und Rehabilitation Nutzen*, *131*, 33–34.
